# Partial Inhibition of Epithelial-to-Mesenchymal Transition (EMT) Phenotypes by Placenta-Derived DBMSCs in Human Breast Cancer Cell Lines, In Vitro

**DOI:** 10.3390/cells13242131

**Published:** 2024-12-23

**Authors:** Yasser Basmaeil, Abdullah Al Subayyil, Haya Bin Kulayb, Altaf A. Kondkar, Maha Alrodayyan, Tanvir Khatlani

**Affiliations:** 1 Stem Cells and Regenerative Medicine Unit, Blood and Cancer Research (BCR) Department, King Abdullah International Medical Research Center (KAIMRC), King Saud Bin Abdulaziz University for Health Sciences (KSAU), Ministry of National Guard Health Affairs (MNGHA), Riyadh 11426, Saudi Arabia; basmaeily@kaimrc.edu.sa (Y.B.); alsubayyilab@ngha.med.sa (A.A.S.); hayabinkulayb@gmail.com (H.B.K.); alrodayyanma@ngha.med.sa (M.A.); 2 Department of Ophthalmology, College of Medicine, King Saud University, Riyadh 11411, Saudi Arabia; akondkar@gmail.com

**Keywords:** placenta, decidua basalis MSCs, MDA231, MCF7, MCF 10A, adhesion, proliferation, migration, invasion, EMT

## Abstract

Stem cell-based therapies hold significant potential for cancer treatment due to their unique properties, including migration toward tumor niche, secretion of bioactive molecules, and immunosuppression. Mesenchymal stem cells (MSCs) from adult tissues can inhibit tumor progression, angiogenesis, and apoptosis of cancer cells. We have previously reported the isolation and characterization of placenta-derived decidua basalis mesenchymal stem cells (DBMSCs), which demonstrated higher levels of pro-migratory and anti-apoptotic genes, indicating potential anti-cancer effects. In this study, we analyzed the anti-cancer effects of DBMSCs on human breast cancer cell lines MDA231 and MCF7, with MCF 10A used as control. We also investigated how these cancer cells lines affect the functional competence of DBMSCs. By co-culturing DBMSCs with cancer cells, we analyzed changes in functions of both cell types, as well as alterations in their genomic and proteomic profile. Our results showed that treatment with DBMSCs significantly reduced the functionality of MDA231 and MCF7 cells, while MCF 10A cells remained unaffected. DBMSC treatment decreased epithelial-to-mesenchymal transition (EMT)-related protein levels in MDA231 cells and modulated expression of other cancer-related genes in MDA231 and MCF7 cells. Although cancer cells reduced DBMSC proliferation, they increased their expression of anti-apoptotic genes. These findings suggest that DBMSCs can inhibit EMT-related proteins and reduce the invasive characteristics of MDA231 and MCF7 breast cancer cells, highlighting their potential as candidates for cell-based cancer therapies.

## 1. Introduction

Breast cancer dominates malignant neoplasms among women worldwide. In 2023, the United States alone reported approximately 300,000 new cases of breast cancer, with an average of 50,000 deaths [1]. Despite technological advances in diagnostics and treatment options, breast cancer still remains a major threat to women’s health, with high medical costs and limited effectiveness of conventional treatments such as surgery, chemotherapy, and radiotherapy [2,3]. As an alternative approach, researchers have been exploring new and non-conventional therapeutic approaches to curb breast cancer [4]. These include small molecules, monoclonal antibodies, cancer vaccines, and cell-based therapies, such as stem cell therapy [5]. Among them, stem cell therapy is accepted as an alternate choice of treatment against breast cancer for its targeted effectiveness and reduced off-target effects [6,7].

With their unique characteristics, including self-renewal, differentiation potential, higher proliferation and migration rates, and immuno-modulatory responses, mesenchymal stem cells (MSCs) make promising cell-based therapeutic agents against various diseases, including cancer [8,9]. MSCs can be isolated from almost all adult tissues, such as adipose tissue, dental pulp, bone marrow, umbilical cord, and placenta [10]. MSCs have been found to exert anti-tumoral responses on cancer tissues that lead to the hindering of disease progression and metastasis. Several studies have shown that this particular phenotype of MSCs is mediated through various processes, including immune modulation, inhibition of angiogenesis, cell cycle regulation, and induction of apoptosis [11]. MSC therapy has been shown to suppress growth of various cancers, such as, breast, glioma, lung, and melanoma, in animal models [12,13,14,15]. In addition, MSC therapy in melanoma mouse models has also demonstrated their pro-apoptotic and anti-angiogenic properties [16]. Furthermore, umbilical cord-derived stem cells have demonstrated apoptosis of GBM cell lines in in vitro studies [17,18]. Finally, MSCs express several cytokines, chemokines, and growth factors with both pro- and anti-apoptotic properties, which makes them suitable tools for targeted cancer therapies [19].

We characterized and investigated the immuno-modulatory properties of MSCs isolated from the decidua part of the human term placenta: *decidua basalis* mesenchymal stem cells (DBMSCs) [20,21]. Like other adult MSCs, DBMSCs express and secrete soluble factors with diverse functions, including immune-modulation, growth inhibition, and apoptosis [20].

To utilize DBMSCs for their effective anti-tumoral responses, it is imperative to first assess their ensuing effects on cancer cell lines. Toward that direction, we hereby investigated the effects of DBMSCs (the cellular component as well as their secretome) on the functional outcome of human breast cancer cell lines MDA-MB-231 (MDA231) and MCF7, and the premalignant-to-malignant breast cancer line MCF 10A, and vice versa. In addition, we evaluated the modulation in expression profiles of selected genes/proteins involved in the process of epithelial-to-mesenchymal transition (EMT).

## 2. Materials and Methods

### 2.1. Cell Lines and Antibodies

MDA-MB-231 (MDA231) Cat# HTB-26, MCF7 Cat# HTB-22, and MCF 10A Cat# CRL-10317 cell lines were purchased from American Type Culture Collection (ATCC, Manassas, VA, USA). Fluorescent-labelled antibodies for flow cytometry experiments, including Human Snail Alexa Fluor^®^ 488-conjugated Antibody, Cat# IC3639G; Human/Mouse/Rat Vimentin APC-conjugated Antibody, Cat# IC2105A; Human Fibronectin PE-conjugated Antibody, Cat# IC1918P; Human/Mouse Wnt-5a Antibody (Cat# MAB645); Human IL-27 PE-conjugated Antibody, Cat# IC25261P; Human BAFF/BLyS/TNFSF13B PE-conjugated Antibody, Cat# IC1241P, Human IL-17RA/IL-17R Antibody; Cat# MAB177; Mouse F(ab)2 IgG (H+L) Fluorescein-conjugated Antibody, Cat# F0103B; Rabbit IgG Fluorescein-conjugated Antibody, Cat# F0112; and Goat IgG (H+L) Fluorescein-conjugated Antibody, Cat# F0109, were purchased from R&D Systems (Minneapolis, MN, USA). Antibodies including IL-21 Monoclonal Antibody (FFA21), PE, Cat# 12-7211-82, and E-Cadherin Monoclonal Antibody (67A4), PE, Cat# A15784, were purchased from Thermo-Fischer Scientific (Waltham, MA, USA). Anti-Interferon Alpha/Beta Receptor 2 Antibody (FITC), Cat# 10359-MM07T-F, was purchased from SinoBiological (Houston, TX, USA).

### 2.2. Placenta and Umbilical Cord Tissue Collection

We collected twenty placentas from healthy pregnant human volunteers within 2 h of delivery of the baby. Only patients/volunteers from whom informed consent and relevant clinical data were available were selected for placenta collection. These studies were submitted as a protocol to the Institutional Review Board (IRB) of King Abdullah International Medical Research Centre (KAIMRC) prior to execution of this study. This study was approved under protocol number RC20/346/R.

### 2.3. Cancer Cell Culture, DBMSC Isolation, Culture, and Conditioned Media (CM) Collection

Cancer cells (MDA231 and MCF7) and MCF 10A cells were cultured in DMEM-F12 medium supplemented with 2% Pen/Strep (ThermoFischer Scientific, Waltham, MA, USA), 10% FBS, and 1% GlutaMax (Gibco^®^, Life Technologies, Riyadh, Saudi Arabia), in T-25 tissue culture flasks, using the standard operating protocols of cell culture.

DBMSCs were isolated from “*decidua basalis*” tissue attached to the maternal side of human placenta, as previously described [20]. Briefly, 10 g of the decidua tissue dissected from the placenta were finely minced after washing and removal of the traces of blood. The minced tissue pellet was re-suspended in digesting solution (0.3% collagenase type I (Life Technologies, Riyadh, Saudi Arabia), 100 μg/mL pen/strep, and 271 units/mL DNase I (Life Technologies, Saudi Arabia)) in PBS, and incubated in a water bath at 37 °C for 1 h. This was followed by filtration, centrifugation, and incubation with red blood cell lysing buffer (FCM Lysing Solution, Cat# sc-3621, Santa Cruz, Riyadh, Saudi Arabia) for 45 min at room temperature. After centrifugation and final washing, 1 × 10^5^ cells were cultured in T-25 flasks in complete DMEM-F12 medium with 10% Mesenchymal Stem Cell Certified Fetal Bovine Serum (MSC-FBS) (Life Technologies, Riyadh, Saudi Arabia), 100 μg/mL L-glutamate, and 100 μg/mL penicillin/streptomycin. Cells were incubated at 37 °C in a humidified chamber and at 75% confluency; the cells were harvested, counted, and re-cultured at a density of 1 × 10^5^ in 75 cm^2^ flasks. At 75% confluency, the cells were harvested, characterized, and used in subsequent experiments, accordingly.

Conditioned medium (CM) from DBMSCs and the cancer cells was collected using our previously standardized protocols [22]. Briefly, 1 × 10^5^ cells were cultured in the appropriate culture medium until they reached a confluency of 75%. Cells were washed, fed with fresh medium, and again incubated for 72 H. The conditioned medium was collected, centrifuged, and stored at −80 °C for future use.

Human Umbilical Vein Endothelial Cells (HUVECs) were isolated from umbilical cord veins using the previously standardized protocols and as described earlier [23]. Briefly, the umbilical veins were flushed with PBS and digested with collagenase type II (Cat# 17101-015, Thermo-Fischer Scientific, Waltham, MA, USA) in PBS solution. The tissue was incubated overnight at 37 °C in a humidified incubator. The released HUVECs were further cultured in complete endothelial cell growth medium (Cat# PCS-100-041™, ATCC, Manassas, VA, USA), for further use.

### 2.4. Inter Cellular Contact (IC) and CM Treatment of Cancer Cells

The cancer cells were treated with DBMSCs using a specialized Transwell chamber that consisted of a membrane with a 0.4 µm pore size. DBMSCs were seeded in the reverse side of the membrane, whereas cancer cells were added to the upper chamber of the membrane in complete medium. For treatment of DBMSCs with the cancer cells, the experimental setup was reversed. Cells were incubated for 24 h, 48 h, and 96 h at 37 °C in a cell culture incubator. The conditioned media (CM) was diluted to various working solutions in complete medium before treatment of the target cells. The treated cells were incubated for various time points (24 h, 48 h, and 96 h) before initiating the functional assays, as described earlier [24].

### 2.5. MTS Assay for Cellular Proliferation

We used an MTS kit (CellTiter 96 Aqueous Non-Radioactive Cell Proliferation Assay, Cat# G5421, Promega, Madison, WI, USA) to assess the proliferation of treated cells and their untreated controls. After treatment, the proliferation of both cancer cells and the DBMSCs was assessed post 24 h, 48 h, and 96 h. Briefly, after incubating the cells in MTS solution for 4 h at 37 °C, their absorbance was recorded at 490 nm using an ELISA plate reader. In order to cease the proliferation of DBMSCs, the cells were treated with mitomycin C (25 μg/mL) at 37 °C for 1 h [22]. Results from three independent experiments are presented as mean ± standard errors.

### 2.6. Real-Time Cell Analysis (RTCA) Assays by xCelligence

xCelligence, the “Real Time Cell Analyzer system” (RTCA-DP version; Roche Diagnostics, Mannheim, Germany), was utilized to assess various cellular functions of both treated and untreated cancer cells as well as the DBMSCs, as per the protocols published earlier [25,26,27].

In order to assess proliferation and adhesion of treated and untreated cells, the “E-Plate 16” (Cat# 05469813001, Roche Diagnostics, Basel, Switzerland) was used according to the manufacturer’s recommendations. Background impedance was recorded before addition of the cells, as previously described [28]. Treated and untreated cells were added to four wells of the plate, which was incubated at room temperature for 30 min before the cells were placed in the xCelligence system, housed in a cell culture incubator. The plates were incubated at 37 °C and the cell indices were monitored consistently for 72 h. While first two hours of the cell index marks the cellular adhesion, the proliferation of the cells was recorded and calculated after 72 h of incubation, when the experiment was terminated. RTCA xCelligence software (version 1.2.1) was used to analyze the data as per the manufacturer’s instructions. Data are expressed as mean normalized cell index with ±standard errors.

For cellular migration of treated and untreated cells, “CIM-16” (Cat# 05665825001, Roche Diagnostics, Basel, Switzerland) plates were used, as described previously [28]. In order to obtain equilibrium, 50 μL of pre-warmed serum-free media were added to the upper chamber of the plate, which was incubated in the RTCA device for 1 H. Treated or untreated control cancer cells at a density of 20 × 10^3^ and in 100 μL culture medium were added to the upper chamber of the plate. The cells were incubated for 30 min at room temperature to attain adherence of cells to the membrane. Complete medium with 10% FBS was added as a chemo-attractant for the migrating cells to the lower chamber of the well. The impedance value was recorded at an interval of 15 min for a total duration of 24 h, when the experiment was terminated.

For assessing the invasion potential of treated and untreated cancer cells, HUVECs were seeded to an E-Plate, as described earlier [19]. Treated and untreated cancer cells in a density of 1 × 10^4^ cells were added to the HUVEC monolayer and the plate was incubated for 48 h. The cellular invasion index was calculated after normalizing the data with the cell index of HUVEC growth.

### 2.7. Cellular Migration and Invasion Assays

The consequences of DBMSCs on the migration and invasion potential of cancer cells were confirmed by Transwell assay. For the migration assay, treated and untreated cells were made to pass through an 8 μm polycarbonate Transwell insert. Similar Transwell inserts with a coating of Matrigel were used for invasion assay. The cells (2.5 × 10^3^ cells/mL) were seeded in the upper chamber of the inserts in serum-free medium, whereas complete medium with 20% FBS was added to the lower chamber of the plate as a chemo-attractant. After incubation of the plates, the migrated/invaded cells were washed, fixed, and stained [29]. The rate of migration and invasion of treated and control cells was determined by normalizing the total number of cells with the migrated/invaded cells for untreated controls.

### 2.8. Real-Time PCR Analysis (RT-PCR)

In RT-PCR, a Human Cytokine and Chemokine RT^2^ Profiler Assay kit (PAHS-150Z; Cat# 330231, Qiagen, Hilden, Germany) was used for DBMSCs treated with CM and a cellular component of cancer cells, and the Human Epithelial to Mesenchymal Transition (EMT) RT^2^ Profiler Assay kit (PAHS-090Z; Cat# 33023 Qiagen, Hilden, Germany) was used for cancer cells treated with DBMSCs to perform the RNA expression profiling

Total RNA was isolated from treated and untreated cells and transcribed into single-stranded cDNA using the Fast Lane cDNA Analysis kit (Cat# 215011, Qiagen, Germantown, MD, USA). In order to detect the expression of a panel of 84 genes, we used the CFX96 real-time PCR detection system (Bio-Rad, Hercules, CA, USA). Data were analyzed by calculating ΔΔ^−2^ values, and are expressed as the fold-change expression compared to the relative expression of genes in the untreated controls. Housekeeping genes (GAPDH and β-actin) were used as internal controls.

### 2.9. Flow Cytometry

A total of 1 × 10^5^ treated and untreated DBMSCs and cancer cells were harvested and stained with fluorescent-labelled antibodies for specific cytokines and chemokines and other proteins involved in EMT, as described earlier and in the “Antibodies and Reagents” section [30]. Briefly, for cell surface expression of proteins, the treated cells were incubated directly with protein-specific antibodies for 30 min, whereas, for intracellular expression, the cells were first fixed with 4% paraformaldehyde and then permeabilized in PBS containing 0.1% saponin. The samples were assayed in a BD FACS CANTO II (Becton Dickinson) flow cytometer. Cells treated with FITC or PE-labelled mouse IgG or IgM antibodies were used as negative control.

### 2.10. Data Analysis

Non-parametric tests, including the Mann–Whitney U test for comparing two groups and the Kruskal–Wallis test for comparing three or more groups, were used. Categorical data were compared using chi-square tests. Data with normal distribution confirmed by Shapiro–Wilk tests were compared using unpaired t-tests for two groups, while ANOVA was applied for comparisons involving more than two groups. Data obtained from three independent experiments are shown in bar and line graphs as means ± standard error (SE). A *p*-value of ≤0.05 was considered statistically significant.

## 3. Results

### 3.1. Standardization of Dose and Temporal Responses

**Figure 1:**
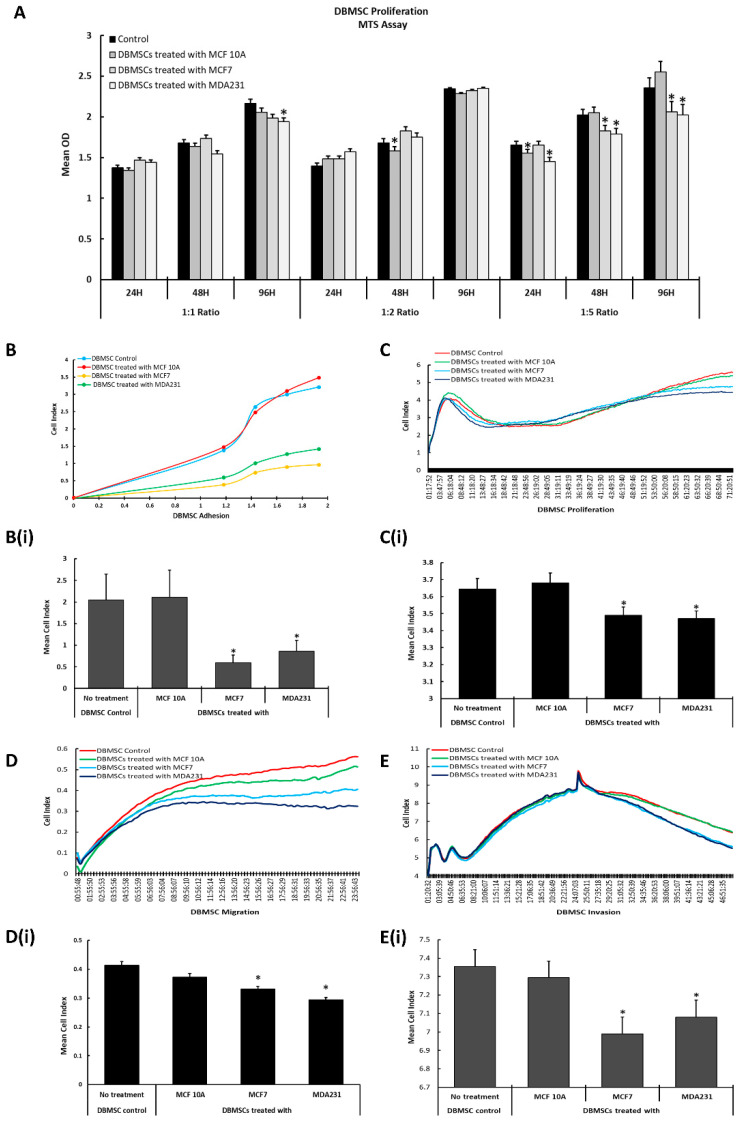
Standardization of cellular ratio and co-culture time for treatment of DBMSCs with cancer cells: the effect of the cellular component of cancer cells at various ratios (1:1; 1:2, and 1:5) and at different time points (24 h, 48 h, and 96 h), with a measurable effect on DBMSCs, as recorded in MTS assay (**A**). Adhesion of DBMSCs decreased significantly after treatment with MCF7 and MDA231 cells for 96 h, but not with MCF 10A cells (**B**,**B(i)**). Proliferation of DBMSCs after co-culture with cancer cells at 1:5 ratio for 96 h decreased significantly with MCF7 and MDA231 cells (**C**,**C(i)**). Similarly, after treatment with the cellular component of MCF7 and MDA231 cells at a 1:5 ratio and incubation for 96 h, DBMSCs showed decreased migration (**D**,**D(i)**), as well as decreased invasion (**E**,**E(i)**), as recorded in the xCelligence assay, compared to MCF 10A and untreated control cells. The experiments were replicated three times with DBMSCs isolated from three different placentas. Bars represent standard errors. * *p* < 0.05.

The appropriate ratio of the cellular component of DBMSCs/cancer cells and the suitable concentration of CM of cancer cells/CM-DBMSCs that have a quantifiable effect on the functional outcome of the DBMSCs/cancer cells was evaluated by MTS assay. We selected three ratios—1:1, 1:2, and 1:5—for DBMSCs to cancer cells, and for CM treatment, the doses were set at 25%, 50%, and 100%, and vice versa. In both settings, the incubation time was set at 24 h, 48 h, and 96 h to calculate the appropriate time where the maximum effect was observed (Figure 1A and Supplementary Figure S1A).

As shown in Figure 1A, DBMSCs treated with MCF7, MDA231, or MCF 10A cells as control at a 1:1 ratio (DBMSC: cancer cells) for 24 h, 48 h, and 96 h did not display any change in their proliferation potential. Similar trends without any significant changes were observed in DBMSCs treated with cancer cells and control cells at 1:2 (DBMSC: cancer cells) cellular ratios for all the time points tested. However, a significant reduction (*p* < 0.05) in proliferation was observed in DBMSCs co-cultured with both MCF7 and MDA231 cells for 48 h and 96 h compared to untreated control (Figure 1A). DBMSCs treated with MCF 10A cells did not exhibit any change in proliferation for any of the time points and CM ratios tested. Based on the results obtained in the MTS assay, a ratio of 1:5 (DBMSCs: cancer cells) and a time point of 96 h was selected for subsequent functional assays.

DBMSCs incubated with CM of cancer cells and well as the control MCF 10A cells for 24 h and 48 h did not show any change in their proliferation, as recorded by MTS assay. However, after 96 h of incubation, they exhibited a decrease in proliferation after treatment with 50% and 100% CM-MDA231. Interestingly, proliferation of DBMSCs increased at 25% and 50% of CM-MCF7 after treatment for 48 h and 96 h (Supplementary Figure S1A). Based on these results, 100% CM of cancer cells with an incubation time of 96 h was selected for subsequent functional assays.

### 3.2. Impact of Cancer Cells on the Functional Capabilities of DBMSCs

Real-Time Cell Analyzer (RTCA) (xCelligence) was used to determine the effect of cancer cells and their CM on the functional outcome of DBMSCs. After sustained co-culture of DBMSCs with MCF7 and MDA231 cells, with MCF 10A as control, for 96 h at a ratio of 1:5, the cells were subjected to cell analysis. Untreated DBMSCs served as control. The cell viability assay of DBMSC-treated cancer cells was estimated at >90% by Trypan blue assay.

DBMSCs co-cultured with MCF7 and MDA231 cells in the IC setting at a 1:5 cellular concentration exhibited significant reduction (*p* < 0.05) in adhesion compared to their treatment with MCF 10A and to the untreated control (Figure 1(B,Bi)). A significant reduction in proliferation was observed in DBMSCs co-cultured with MCF7 and MDA231 cells and not in those treated with MCF 10A cells and in untreated control DBMSCs, as shown in Figure 1(C,Ci). Similarly, the cellular content of MCF7 and MDA231 cells, and not the MCF 10A cells and the untreated control, decreased the migration and invasion potential of the DBMSCs at 1:5 cellular ratios significantly (*p* < 0.05) when co-cultured for 96 h and tested in xCelligence assay (Figure 1(D,Di,E,Ei)).

DBMSCs incubated with 100% CM of all the cancer cells or MCF 10A cells and tested for 96 h did not change their adhesion potential (Supplementary Figure S1(B,Bi)). Although treatment of DBMSCs with the CM of MCF7 cells decreased the proliferation of DBMSCs compared to the CM of MDA231 and MCF 10A cells, the decrease was not statistically significant (Supplementary Figure S1(C,Ci)). Similarly, the CM of the cancer cells did not change the migration and invasion potential of the DBMSCs (Supplementary Figure S1D,Di,E,Ei)).

**Figure 2:**
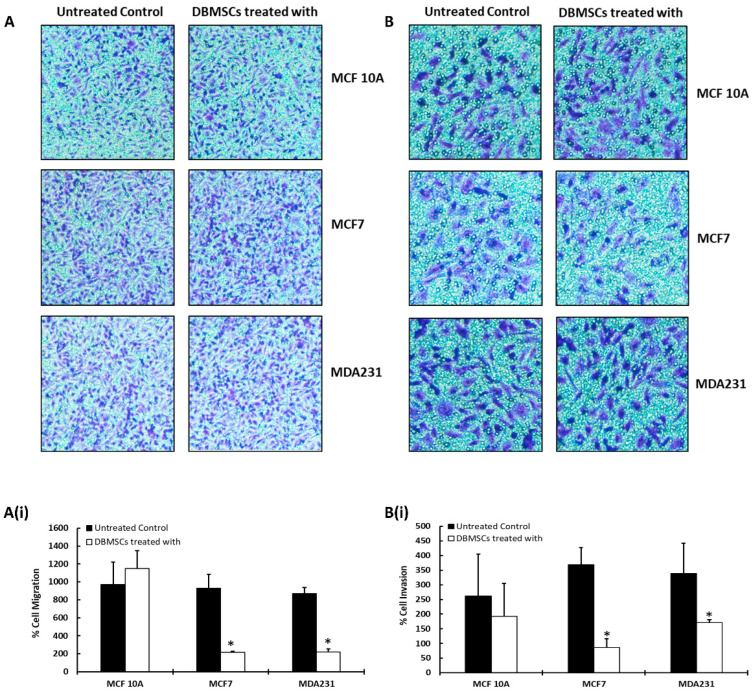
Migration and invasion of DBMSCs after their co-culture with cancer cells: migration and invasion of DBMSCs after their treatment for 96 h with the cellular component of MCF 10A, MCF7, and MDA231 cells at a 1:5 ratio, further evaluated by Transwell assay. DBMSCs treated with MCF7 and MDA231 cells migrated at a significantly faster rate through the 8 µM pore of a Transwell filter compared to the MCF 10A cells and the untreated control. Panel (**A**) shows the photomicrograph (20X magnification) of the migrated cells under various treatment conditions, whereas the percentage of total migrated cells is presented in a bar graph (**A(i)**). Similar results were observed for the invasion assay performed over Matrigel-coated Transwell plates under similar treatment and incubation conditions (**B**,**B(i)**). DBMSCs isolated from three individual placentas were used in these experiments. The experiments were repeated three times for statistical analyses. Bars represent standard errors. * *p* < 0.05).

The migratory and invasive potential of the DBMSCs after treatment with cellular components of cancer cells were further verified by Transwell assay. As shown in Figure 2, the co-culture of DBMSCs with MCF7 and MDA231 cells exhibited a significantly reduced (*p* < 0.05) number of cells that had migrated through the membrane (Figure 2A,A(i)) compared to the untreated control. However, treatment of DBMSCs with 100% CM of cancer cells (MCF7 and MDA231) for 96 h, followed by subsequent Transwell assay, did not demonstrate a significant change in the rate of their migration (Supplementary Figure S2A,A(i)). These results are in accordance with the results obtained in the xCelligence RTCA assays.

Compared to the untreated control and treatment with MCF 10A cells, the invasion of DBMSCs changed significantly (*p* < 0.05) in the Transwell assay, as shown in Figure 2B,B(i). However, DBMSCs incubated with 100% CM of MCF7 and MDA231 cells did not exhibit any significant reduction in invasion across the Matrigel-coated membrane (Supplementary Figure S2B,B(i)), indicating that the CM of the cancer cells did not alter the invasive potential of DBMSCs.

### 3.3. Modulation in Expression of Anti-Tumor and Pro-Apoptotic Molecules in DBMSCs in Response to Cancer Cell Treatment

Expression of the essential factors responsible for modulation of functional characteristics of DBMSCs, after co-culture with cancer cells, was evaluated by RT-PCR analysis and verified by flow cytometry. The Cytokine and Chemokine RT^2^ Profiler™ PCR Array kit was utilized to quantify the expression of proteins in DBMSCs with anti-cancer and pro-apoptosis properties. Table 1 illustrates the modulation in expression of numerous molecules with tumor-suppressor or oncogenic properties in DBMSCs after co-culture with the cellular component of MDA231 cells. Various genes with tumor suppressor functions, such as chemokine ligand-11, IL-21, IL-27, CD40L, chemokine ligand-9, IL-9, and IL-15, were upregulated in DBMSCs after treatment with MDA231 cells in an IC setting. In addition, several molecules, including CSF3, chemokine ligand-22, BMP2, IL-13, and IL-17 with oncogenic properties, were downregulated in DBMSCs after treatment with MDA231 cells in an IC setting.

**Figure 3:**
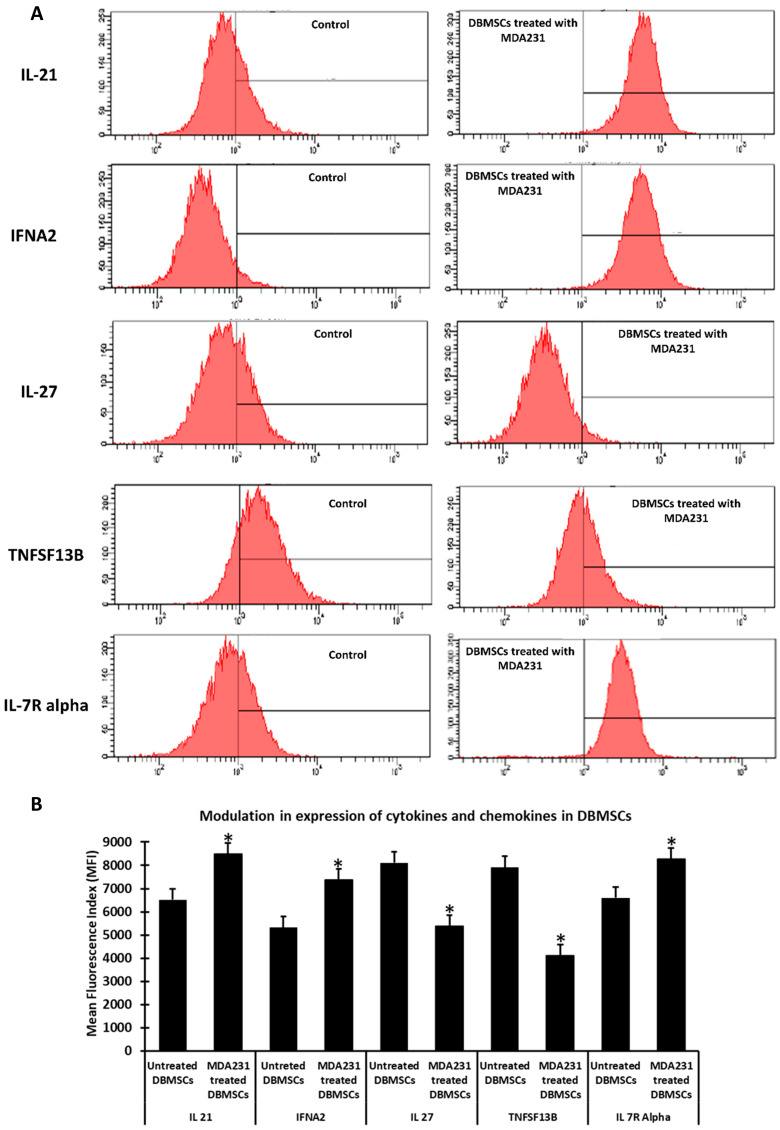
Effect of cancer cell treatment on the expression of chemokines and cytokines on DBMSCs: Flow cytometry analysis for expression of chemokines and cytokines by DBMSCs after co-culture with DBMSCs showed a significant increase in IL-21, IFNA2, TNSF13B, and IL-17R for 96 h (**A**) but a significant decrease in the expression level of IL-27. The mean fluorescence index (MFI) is plotted and shown as a bar diagram (**B**). Bars represent standard errors. * *p* < 0.05.

Modulation in expression of these effector molecules was further assessed at the proteomic levels. In the flow cytometry assay, the expression of IL-21, IL-27, and IFNA2 proteins with anti-tumor functions were elevated significantly in DBMSCs after treatment with MDA231 cells compared to the untreated DBMSCs (Figure 3A). Similarly, expression of oncogenes such as TNFSF1B and IL7R alpha decreased significantly in DBMSCs co-cultured with MDA231 cells compared to the untreated DBMSCs (Figure 3A) in the IC setting. Figure 3B illustrates the relative expression of anti-tumor and oncogenic proteins in DBMSCs treated with MDA231 cells compared to the untreated DBMSC controls. Expression of these selected proteins were modulated significantly (*p* < 0.05) in MDA231-treated DBMSCs in the co-culture setting. Expression of these molecules was recorded as mean fluorescence index (MFI) units.

### 3.4. Impact of DBMSCs on the Functional Capabilities of Cancer Cells

In order to utilize the DBMSCs for anti-cancer therapies, it is necessary to evaluate their effect on the functional capabilities of cancer cells. We utilized the MTS and xCelligence assays to measure the effect of the cellular component of DBMSCs and their CM over cancer cells, including MCF7, MDA231, and control MCF 10A cells.

**Figure 4:**
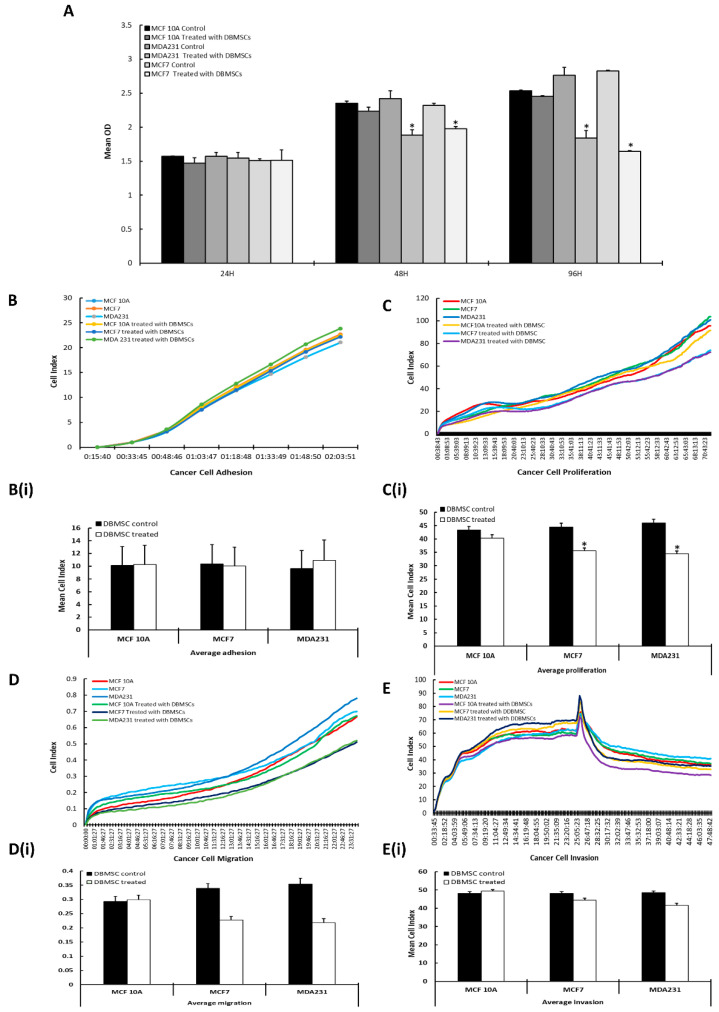
Standardization of co-culture time for treatment of cancer cells with DBMSCs: the effect of the cellular component of DBMSCs at different time points (24 h, 48 h, and 96 h) with a measurable effect on MCF 10A, MCF7, and MDA231 cells, as recorded in the MTS assay (**A**). Adhesion of all cancer cells as well as the control cells did not change after treatment with DBMSCs for 96 h (**B**,**B(i)**). Proliferation of MCF7 and MDA231 cells decreased significantly after co culture with DBMSCs at a 1:5 ratio for 96 h (**C**,**C(i)**). Similarly, after treatment with the cellular component of DBMSCs at a 1:5 ratio, and after incubation for 96 h, MCF7 and MD231, but not MCF 10A or the untreated controls, showed decreased migration (**D**,**D(i)**) as well as decreased invasion (**E**,**E(i)**), as recorded in the xCelligence assay. The experiments were replicated three times with DBMSCs isolated from three separate placentas. Bars represent standard errors. * *p* < 0.05.

After sustained incubation of cancer cells for 24 h, 48 h, and 96 h with DBMSCs at a ratio of 1:5 (cancer cells: DBMSCs) in IC settings, and at 25%, 50%, and 100% concentrations of CM-DBMSCs, the cancer cells were subjected to cell analysis by MTS assay. MCF 10A and untreated cancer cells served as control. The cell viability of DBMSC-treated cancer cells was found at ~95%. Cancer cells treated with DBMSCs at 24 h did not show any change in their proliferation. However, IC treatment of MCF7 and MDA231 by DBMSCs for 48 h and 96 h showed a significant decrease (*p* < 0.05) in cancer cell proliferation. No such change was observed in MCF 10A cells or the untreated controls (Figure 4A). Since the change in proliferation was significantly robust in cells treated under sustained IC conditions for 96 h (Figure 4A), this time point was chosen to study the functional consequences of cancer cells post treatment of DBMSCs.

As shown in Figure 4B, although a subtle increase in adhesion of cancer cells was observed after their treatment with DBMSCs at the ratio of 1:5 in IC settings in the xCelligence RTCA assay, the increase was not statistically significant (Figure 4B(i)).

Both MDA231 and MCF7 cells demonstrated a significant (*p* < 0.05) decrease in proliferation, measured after 96 h co-culture with DBMSCs, compared to untreated control and MCF 10A cells, in the xCelligence RTCA system (Figure 4C). The growth curves corresponded to the normalized cell indices calculated for all experimental groups, where the cancer cells showed a significant decrease in proliferation in the IC settings when treated with DBMSCs for 96 h (Figure 4C(i)).

Cancer cell migration decreased significantly following the IC treatment with DBMSCs, as shown in Figure 4D. No significant changes were observed in MCF 10A cells treated with DBMSCs or the untreated controls. The data correlated with the mean cell index evaluated for all experimental replicates, where DBMSCs treatment in the IC setting exhibited a significant (*p* < 0.05) decrease in the migration of MCF7 and MDA231 cells (Figure 4D(i)).

DBMSC treatment of MCF7 and MDA231 cells in IC settings reduced their invasive potential, as observed in the xCelligence assay (Figure 4E). The mean cell index of all the experiments performed was corroborated with the findings of the xCelligence assay, with a significant reduction (*p* < 0.05) in invasion, as shown in (Figure 4E(i)).

**Figure 5:**
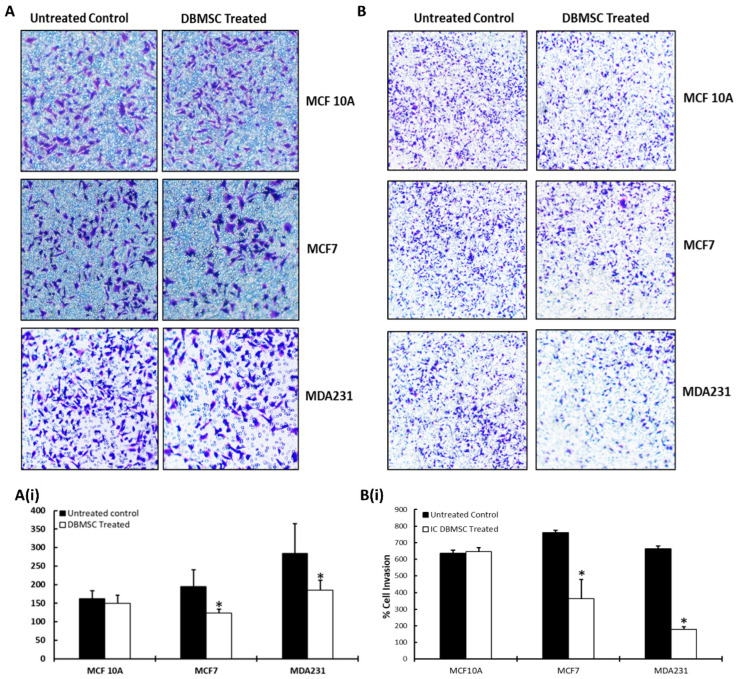
Migration and invasion of cancer cells after their treatment with DBMSCs: MCF7 and MDA231 migration and invasion after their treatment with DBMSCs at a 1:5 ratio and for 96 h were further assessed by Transwell assays. MCF7 and MDA231 cells migrated at a significantly slower rate through the 8 µM pore of a Transwell membrane after their treatment with the cellular component of DBMSCs for 96 h at a 1:5 ratio, compared to the MCF 10A cells and untreated controls. Panel (**A**) shows the photomicrographs (20X magnification) of the migrated cancer cells, whereas the percentage of total migrated cells is presented in a bar graph (**A(i)**). Similar results were observed for an invasion assay performed over a Matrigel-coated Transwell membrane under similar treatment and incubation conditions (**B**,**B(i)**). DBMSCs isolated from three different placentas were used in these experiments. The experiments were repeated three times for statistical analyses. Bars represent standard errors. * *p* < 0.05).

The modulation in migration and invasion potential of cancer cells and the control MCF 10A cells after treatment with DBMSCs was verified by the Transwell assay. The cancer cells and the control cells were co-cultured with DBMSCs for 96 h in IC settings, followed by passage through an 8 µM porous membrane for migration assay. Membranes coated with Matrigel and with the same pore size were used in an invasion assay. Figure 5A shows that co-culture of MCF7 and MDA231 cells with DBMSCs exhibited a significant reduction (*p* < 0.05) in the number of cells that migrated through the membrane compared to the untreated control and MCF 10A cells. Figure 5A(i) shows the actual count of cells presented as a percentage of migration through the Transwell membrane. These results are in accordance with the findings of the xCelligence RTCA assay.

As shown in Figure 5B, compared to the control MCF 10A cells, the invasion of both MCF7 and MDA231 cells changed significantly after their co-culture with the cellular component of DBMSCs. Figure 5B(i) shows the actual number of cells, presented as a percentage of invaded cells, through the Transwell membrane. These results are in agreement with the findings of the xCelligence RTCA assay and are statistically significant (*p* < 0.05).

### 3.5. Modulation in Expression of EMT Effectors in Cancer Cells in Response to DBMSC Treatment

Modulation in the expression of genes responsible for EMT in cancer cells after their co-culture with DBMSCs was analyzed by RT-PCR and verified by flow cytometry analysis. The RT^2^ Profiler™ PCR Array Human Epithelial to Mesenchymal Transition kit was used to evaluate modulation in gene expression. Expression of several genes regulating EMT either positively or negatively was found to be modulated after co-culture of DBMSCs with the cancer cell line MDA231 (Table 2). Molecules with tumor suppressor functions such as CAMK2N1, CDH1, IGFBP4, RGS2, and WNT5A were upregulated in MDA231 cells after their co-culture with DBMSCs in an IC setting. In addition, several molecules, including AKT1, CALD1, GSK3B, NOTCH1, and SNAI1, were downregulated in MDA231 cells after co-culture with DBMSCs in an IC setting.

**Figure 6:**
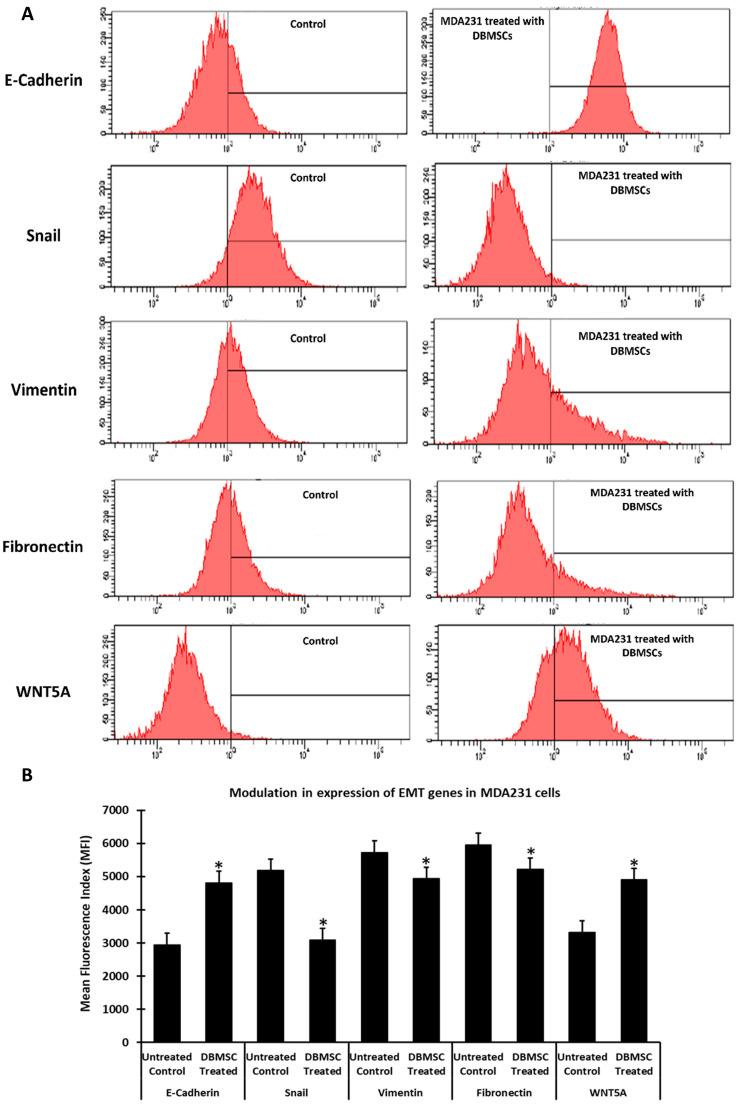
Effect of DBMSC treatment on the expression of EMT effectors on MDA231 cells: Flow cytometry analysis for the expression of effectors responsible for EMT by MDA231 cells after co-culture with MDA231 cells for 96 h showed a significant increase in E-cadherin and WNT5A (**A**), and a significant decrease in the expression level of Snail, vimentin and fibronectin. The mean fluorescence index (MFI) is plotted and shown as a bar diagram (**B**). Bars represent standard errors. * *p* < 0.05.

Differential RNA expression of genes in MDA231 cells, responsible for EMT were further investigated at proteomic levels by flow cytometry. Figure 6A shows the changes in expression pattern of important effector molecules in DBMSCs treated MDA231 cells, such as CDH1, SNA1, VIM1, FBN and WNT5A as compared to the untreated controls. Expression levels of E-Cadherin and WNT5A increased significantly, whereas the levels of Snail 1, Vimentin and Fibronectin decreased significantly (*p* < 0.05) in DBMSC treated MDA231 cells in IC settings as compared to untreated control (Figure 6B).

These data suggest that the cellular component of the DBMSCs alters EMT potential of cancer cells in vitro.

## 4. Discussion

In mesenchymal stem cells isolated from the decidua region of human term placenta, DBMSCs have peculiar characteristic features that render them potentially useful for anti-cancer therapies [20,79,80,81]. DBMSCs are multipotent cells that differentiate into multiple lineages and possess immunomodulatory properties. They secrete and express several important molecules that modify the functional activities of their target cells [82]. Most importantly, these cells express and secrete a distinct combination of cytokines, chemokines, growth factors, and immune-modulating molecules, reflecting their uniqueness [20]. We have previously reported that DBMSCs induced stimulatory effects on CD4^+^ T-cell proliferation, leading to the secretion of several pro-inflammatory molecules. In addition, DBMSCs enhanced the differentiation of M1-like inflammatory macrophages, which function as anti-tumor cells [83]. Furthermore, DBMSCs have the potential to protect endothelial cells from activation, thereby inhibiting endothelial proliferation, migration, adhesion, and expression of inflammatory markers [84]. Based on these exceptional properties, DBMSCs could be attractive, alternative candidates for MSC-based therapies against cancer.

Inflammatory diseases, including cancer, are characterized by high levels of inflammatory and oxidative stress mediators in their microenvironment, which lead to inactivation of cell-based therapies [85,86,87]. In order to utilize DBMSCs for successful cancer therapies, they must withstand the harsh and toxic environment for their survival before exhibiting their anti-tumor properties. Therefore, it was imperative to first study their functional and physiological characteristics post exposure to a system mimicking the cancer environment.

In this study, we first investigated the impact of the cellular component and the secretome of the breast cancer cell lines MDA231 and MCF7 and the control cell line MCF 10A on the functional consequences of DBMSCs. The phenotypic and genotypic modulations of DBMSCs after cancer cell treatment were evaluated. The appropriate number of cancer cells and the specific dose of their conditioned media, having a quantifiable outcome over the performance of DBMSCs, was necessary to measure. The spatiotemporal effects on DBMSCs were measured after their treatment with the cellular component, or with the specific ratio of the CM, for a specific time period. This was followed by measuring various physiological and functional parameters.

With a significant decrease in the proliferation potential, DBMSCs survived the toxic environment of cancer, as observed in Figure 1A,C,C(i). The decrease in their proliferation potential may be attributed to the secretion of several inflammatory molecules, such as IL-1β, IL-6, IL-8, IL-10, and matrix metallo-proteinases (MMPs) by cancer cells, which have adverse effects on the neighboring cells in the niche. It has been reported that expression of these molecules causes cellular injury and apoptosis in MSCs and other cells, including pancreatic beta cells [88,89,90,91]. MDA231 and MCF7 cells secrete anti-proliferative proteins, including IL-10, plasminogen activator inhibitor type 1 (PAI-1), and parathyroid hormone-related protein (PTHrP) [89,92,93,94,95], which may also modulate the proliferative potential of DBMSCs. DBMSCs are isolated from the maternal side of the placental niche, which is rich in oxidative stress markers and pro-inflammatory mediators. Survival, maintenance of their functional abilities, and resistance to apoptosis induced by inflammatory molecules in such a toxic environment may be associated with their acquired biological phenotypes, which they attain during the pregnancy period [96,97]. The reduction in the proliferation of DBMSCs in the tumor setting suggests that there will be fewer chances of teratoma formation, as previously reported for other types of MSCs. It was shown that the transplantation of human bone marrow-derived MSCs (BMMSCs) does not form malignant tumors [98]. Therefore, the use of DBMSCs as a therapeutic agent is probably safe but requires further confirmation in in vivo animal models before their applications in human subjects.

The ability of DBMSCs to maintain their functional activities after injury induced by cancer cells and their secretome was further supported by subsequent functional assays. Treatment of DBMSCs with MDA231 and MCF7, but not with MCF 10A, reduced their adhesion potential (Figure 1B,B(i)). It has been reported that IL-10 secreted by cancer cells is involved in reduction in cellular adhesion, and its expression by breast cancer cells may modulate a decrease in the adhesion potential of DBMSCs [99].

The cellular content of MDA231 and MCF7 cells inhibited the migration and invasion potential of DBMSCs, as opposed to the MCF 10A treated and untreated control cells (Figure 1D,D(i),E,E(i) and Figure 2A,B, respectively). This phenomenon may be related to modulation in the expression of several chemokines and cytokines by DBMSCs under the influence of cancer cells. These include IL-27, IFNA2, CXCL10, IL-21, TNFSF1B, IL-7R, CSF3, and CCL22 (Table 1 and Figure 3A,B). Modulation in the expression of these genes has been reported to be responsible for changes in several cellular functions, including cellular adhesion, cellular proliferation, migration, and invasion [33,39,40,42,43]. In addition to modulation in expression of chemokines and cytokines under the influence of cancer cells, it has been reported that DBMSCs express several other important molecules, which have a significant impact on the target cells in the transplanted niche [20]. These include molecules with anti-cancer properties, which have been reported to modulate several cellular functions in other placenta-derived MSCs, such as DPMSCs and CVMSCs [22,29]. In conclusion, this study shows that DBMSCs retain stem cell properties and are functionally viable in the cancer setting. Furthermore, they are capable of delivering anti-tumor properties effectively and safely in the tumor microenvironment.

Next, we investigated the impact of DBMSCs on the functional capabilities of human breast cancer cell lines MDA231, and MCF7. Phenotypic and genotypic modulations of DBMSC-treated cancer cells were also evaluated. In order to evaluate the differential impact of DBMSCs on cancer and normal tissues, our studies clearly indicate that various concentrations of CM or different cellular ratios of DBMSCs did not alter the survival of MCF 10A cells.

On the contrary, co-culture of MDA231 and MCF7 cells with DBMSCs in the IC setting as well as their treatment with 100% CM of DBMSCs (Supplementary Figure S3) minimized their viability, as shown in Figure 4A. MSCs secrete various essential factors, including chemokines, cytokines, and growth factors, that exert their specific effect on the target cells, leading to tissue regeneration, immune modulation, and anti-apoptosis [100]. As reported above, cancer cells modulate the expression and secretion of several anti-proliferative molecules on DBMSCs (Table 1), including, CD40LG, CXCL9, IL9R, IL-15, TNFSF13B, IL-9, IL-17, and CXCL1 [35,36,38,47,52,53,101]. The combined activities of these molecules may be responsible for suppression of the proliferative potential of cancer cells when treated with DBMSCs in IC settings, as seen in Figure 4A,C,C(i).

The differential impact of DBMSCs on cancer cells and the control MCF-10A cells may be based on the differential expression of novel and unidentified molecules in DBMSCs that build up a conducive environment for DBMSCs to exert a selective and distinct effect on each of the cell types. It is speculated that MCF 10A cells did not produce any toxic environment when treated with DBMSCs and thus, no change in cellular viability was observed, as depicted in Figure 4A,C,C(i). Similar results were obtained when cancer cells and the control MCF 10A cells were treated with different doses of CM of the DBMSCs under similar conditions. Further investigation is needed to identify the unidentified and novel target molecules that are differentially expressed by the DBMSCs under the influence of cancer cells and the control MCF 10A cells.

The significant decrease in proliferation of cancer cells after treatment with DBMSCs supports the postulation that MSC treatment may modulate their other functional parameters. The inhibition of adhesion molecules led to the detachment of cancer cells from their primary site and into the lymphatic or blood stream that carries them to distant sites. These detached cells adhere, proliferate, and result in a de novo tumor, a process called metastasis [102]. Cellular adhesion of cancer cells with their extracellular matrix plays a central role in tissue integrity and architecture. This process regulates other cellular functions, including cellular proliferation, migration, and invasion, leading to regulation of the metastasis of various cancers [103]. No significant change in cancer cell adhesion was observed after co-culture with DBMSCs in the CM setting, as opposed to the untreated control (Supplementary Figure S3B,B(i)). However, treatment with the cellular component of DBMSCs in the IC setting showed a subtle decrease in cancer cell adhesion, as shown in Figure 4B,B(i). The treatment of cancer cells with DBMSCs in the IC setting modulated the expression of various factors with a specific role in cellular adhesion, including AKT1, CALD1, CDH1, FN1, and STAT3 [58,59,64,65,74,104]. Their definite role in the modulation of cancer cell functions after treatment with DBMSCs needs to be verified independently. We reported earlier that DPMSCs also secrete pro-adhesive molecules in the cancer microenvironment through their paracrine effect, which regulates the adhesive properties of MDA231 cells [22].

Proliferation of cancer cells but not the MCF 10A cells decreased significantly after their co-culture with DBMSCs in the IC setting as well as after their treatment with the secretome of the stem cells, as shown in Figure 4C,C(i) and Supplementary Figure S3C,C(i). This is in agreement with our previous studies, where mesenchymal stem cells isolated from different parts of the placenta, such as DPMSCs and CVMSCs, showed a significant reduction in proliferation of MDA231 cells in vitro [19,105]. It has also been reported that MSCs isolated from umbilical cord tissue reduce proliferation and induce apoptosis in human glioma cell lines [106]. Furthermore, co-culture of MSCs isolated from several other sources inhibit tumor growth and proliferation of breast [107,108,109], lung [106] colorectal [106,110], brain [18,106,111,112,113], esophageal [109], and ovarian cancers [114].

Metastasis is associated with poor survival and increased chances of mortality in cancer patients [115]. Cancer cells after detachment from the primary tumor have the potential to utilize their migratory and invasive properties, leading to metastasis at distant sites [116,117]. Co-culture of cancer cells with DBMSCs resulted in a significant decrease in their migration and invasion potentials (Figure 4D,D(i),E,E(i) and Supplementary Figure S3D,D(i),E,E(i)). Genomic analysis revealed that DBMSC treatment of cancer cells modulated several genes responsible for cellular migration and invasion. Upregulation of several genes, including IGFBP4, RGS2, and WNT5A, that mediate several functions, including growth, differentiation, proliferation, apoptosis, and angiogenesis [55,56,57], were observed in cancer cells after their treatment with DBMSCs. Similarly, several effector molecules, including CALD1, CDH1, FN1, FZD7, RAC1, STAT3, and WNT11, were downregulated in cancer cells treated with DBMSCs. These molecules are responsible for the growth, migration, invasion, angiogenesis, and metastasis of cancer cells [58,60,64,65,71,74,77]. This is in agreement with our previous findings, where DPMSCs modulated the expression of several genes in cancer cells that play significant roles in cellular migration and invasion [19]. It has been documented that MSCs derived from human cord blood minimized the invasion and migration potential of glioblastoma cell lines by downregulating c-Myc/ERK, PI3K/AKT, and EGFR/c-Met pathways [112]. Furthermore, it has been reported that Wnt signaling modulated cellular migration of breast cancer cells by MSCs from human umbilical cord tissues and adipose tissues [118,119].

Cancer metastasis starts with morphological changes from a compact to a more expanded phenotype, followed by detachment and migration, and finally settling at a distant site. The process of losing the cellular polarity and cell–cell adhesion, and transitioning from an immotile (epithelial) to a motile (mesenchymal) phenotype, is known as epithelial-to-mesenchymal transition (EMT), which correlates with disease progression and tumor metastasis. In EMT, the benign tumor cells lose their adhesive properties and gain migratory and infiltrative properties as the tumor progresses [120]. The vast majority of tumors, including prostate, lung, liver, pancreatic, and breast cancers, have been demonstrated to undergo EMT during tumor progression [121,122]. EMT involves the modulation of genes responsible for maintaining the mobility of epithelial cells and those involved in their migration and invasion [120,123]. Modulation in the expression of various genes responsible for EMT was observed in both MCF7 and MDA231 cells after their co-culture with DBMSCs (Table 2). Among them, upregulated genes such as CAMK2N1, IGFBP4, RGS2, WNT5A, and CDH1 have both tumor suppressor and inhibitory effects on EMT [54,55,56,57,58]. It has been demonstrated that Snail1 induces EMT by repressing E-cadherin, leading to reduced cellular adhesion and promoting migration [124].

Proteins involved in various adherin junctions and cytoskeleton reorganization, including N-cadherin, β-catenin, and vimentin, are regulated by SNAIL1 and ZEB1 in a coordinated fashion. In addition, it has been demonstrated that TGF-β and STAT3 pathways are the strongest inducers of EMT [125,126]. Treatment of cancer cells, including MDA231 and MCF7 cells, with a cellular component of DBMSCs in an IC setting, demonstrated downregulation of various effector molecules, including AKT1, CALD1, FN1, FZD7, SNA1, MMP2 and 7, STAT3, TGFB1, and vimentin, that play crucial roles in the initiation of EMT phenotypes in various cancers [59,60,61,64,69,70,73,74,75,76]. Modulation in the expression of EMT-related genes was further confirmed at proteomic levels by flow cytometry, the results of which are in agreement with the data obtained in the genomic analysis by RT-PCR (Figure 6).

## 5. Conclusions

The overall results of this study suggest that DBMSCs induce a loss of the proliferative, migratory, and invasive potentials of breast cancer cell lines in vitro. Most importantly, treatment with DBMSCs induces upregulation of markers that are involved in the inhibition of EMT phenotype and downregulate the markers that induce EMT in breast cancer. These results suggest that DBMSCs may be an important addition as a source of MSCs to be utilized in the field of cellular therapy against cancer. However, detailed in vitro and in vivo studies need to be executed to validate their mechanism of action, efficacy, and safety, prior to human applications.

## Figures and Tables

**Table 1:** Modulation in chemokine and cytokine expression in DBMSCs after treatment with MDA231 cells.

Gene ID	Gene Name	Biological Function	Expression Levels	References
CXCL11	Chemokine (C-X-C motif) ligand 11	Promotes antitumor immunity.	4.7571	[31]
IL21	Interleukin 21	Provokes anti-tumor responses in solid cancers.	3.1631	[32]
IFNA2	Interferon, alpha 2	Decreases proliferation, migration, and invasion.	1.6112	[33]
CD40LG	CD40 ligand	Potentiates apoptosis of tumor cells.	2.8939	[34]
CXCL9	Chemokine (C-X-C motif) ligand 9	Inhibits tumor growth.	1.6350	[35]
IL9R	Interleukin 9 receptor	Involved in anti-tumor immunity.	4.1763	[36]
CCL17	Chemokine (C-C motif) ligand 17	High expression leads to survival of patients.	6.9612	[37]
IL9	Interleukin 9	Anti-cancer function in solid tumors, promotes apoptosis.	3.0494	[36]
IL15	Interleukin 15	Preserves the CAR-T cell phenotype and antitumor activity.	1.0620	[38]
IL 27	Interleukin 27	Involved in anti-tumor and anti-proliferative activities.	4.6179	[39]
CXCL10	Chemokine (C-X-C motif) ligand 10	Involved in induction of apoptosis and cell growth.	5.3684	[40]
C5	Complement component 5	Associated with better survival.	5.2924	[41]
CSF3	Colony stimulating factor 3 (granulocyte)	Promotes carcinoma cell proliferation and migration.	0.2602	[42]
CCL22	Chemokine (C-C motif) ligand 22	Enhances proliferation and invasion and inhibits apoptosis.	0.3269	[43]
CXCL2	Chemokine (C-X-C motif) ligand 2	High expression is involved in invasion and metastasis.	0.7589	[44]
BMP2	Bone morphogenetic protein 2	Induces EMT and breast cancer stemness.	0.8667	[45]
LTA	Lymphotoxin alpha (TNF superfamily, member 1)	High expression is involved in survival.	0.8030	[46]
TNFSF13B	Tumor necrosis factor (ligand) superfamily, member 13b	Supports cell proliferation and suppresses apoptosis.	0.8198	[47]
IL13	Interleukin 13	Promotes EMT and aggressiveness of CRC.	0.1823	[48]
IL17C	Interleukin 17C	Associated with autoimmune disorders and cancers.	0.7802	[49]
CXCR2	Chemokine (C-X-C motif) receptor 2	Involved in tumor regulation and growth.	0.0949	[50]
CCL4	Chemokine (C-C motif) ligand 4	Facilitates pro-tumorigenic capacities.	0.2638	[50]
CXCL13	Chemokine (C-X-C motif) ligand 13	Contributes to lung carcinogenesis.	0.7047	[51]
IL7	Interleukin 17	Associated with tumor development.	0.1596	[52]
CXCL1	Chemokine (C-X-C motif) ligand 1	Associated with loss of tumor suppressors.	0.9077	[53]

Modulation of chemokine and cytokine expression in DBMSCs after their co-culturing with MDA231 cells: differential gene expression observed in DBMSCs after treatment for 96 h with MDA231 cells and normalized with the untreated control. RT-PCR was performed using the Chemokine and Cytokine RT^2^ Profiler kit (Qiagen), as explained in the “Materials and Methods” section. Three independent experiments were performed, and data are calculated as fold change increase or decrease, calculated from the ΔΔ^−2^ values, and expressed as upregulated or downregulated in the table.

**Table 2:** Modulation in expression of EMT genes in MDA231 cells after treatment with DBMSC cells (IC).

Gene ID	Gene Name	Biological Function	Expression Levels	References
CAMK2N1	Calcium/calmodulin-dependent protein kinase II inhibitor 1	Regulates glioma cell proliferation and apoptosis.	5.5929	[54]
IGFBP4	Insulin-like growth factor binding protein 4	Blocks IGF activity, leading to inhibition of tumor growth.	4.9675	[55]
RGS2	Regulator of G-protein signaling 2	Suppresses breast cancer cell growth.	5.3590	[56]
WNT5A	Wingless-type MMTV integration site family, member 5A	Induces tumor suppression in breast cancer patients.	6.0165	[57]
CDH1	Cadherin 1, type 1, E-cadherin (epithelial)	Inhibits EMT, tumor proliferation, invasion, and migration.	0.6475	[58]
AKT1	V-akt murine thymoma viral oncogene homolog 1	Negative regulator of EMT and metastasis.	0.9241	[59]
CALD1	Caldesmon 1	Promotes growth, migration, and invasion, and inhibits apoptosis.	0.5243	[60]
CDH2	Cadherin 2, type 1, N-cadherin (neuronal)	Positively regulates EMT, cellular proliferation, and metastasis.	0.9498	[61]
COL1A2	Collagen, type I, alpha 2	Abnormal expression in gastric and pancreatic cancers.	0.8869	[62]
FGFBP1	Fibroblast growth factor binding protein 1	Involved in angiogenesis during cancer progression.	0.1763	[63]
FN1	Fibronectin 1	Promotes cellular proliferation and migration in gastric cancer.	0.5709	[64]
FZD7	Frizzled family receptor 7	Involved in cancer growth, metastasis, and chemo-resistance.	0.0497	[65]
GNG11	Guanine nucleotide binding protein, gamma 11	High expression indicated poor prognosis in ovarian cancer.	0.4444	[66]
GSK3B	Glycogen synthase kinase 3 beta	Contributes to the pathogenesis and progression of cancer.	0.6873	[67]
ITGAV	Integrin, alpha V	Associated with poor survival of breast cancer patients.	0.4945	[68]
MMP2	Matrix metallopeptidase 2	Associated with poor prognosis in bladder and ovarian cancer.	0.3106	[69]
MMP9	Matrix metallopeptidase 9	Associated with poor prognosis in bladder and ovarian cancer.	0.8559	[69]
NOTCH1	Notch 1	Promotes epithelial–mesenchymal transition (EMT).	0.3887	[70]
RAC1	Ras-related C3 botulinum toxin substrate 1	Highly expressed in proliferating breast cancer.	0.3229	[71]
SERPINE1	Serpin peptidase inhibitor, clade E	Exhibits aberrant expression in various cancers.	0.2695	[72]
SNAI1	Snail homolog 1	Master transcription factors of EMT and a metastatic marker.	0.2292	[73]
STAT3	Signal transducer and activator of transcription 3	Affects breast cancer progression, proliferation, and metastasis.	0.1909	[74]
TGFB1	Transforming growth factor, beta 1	Enhances the tumorigenicity and invasiveness of breast cancer.	0.0530	[75]
VIM	Vimentin	Promotes EMT, aggressiveness, and resistance to chemotherapy.	0.0195	[76]
WNT11	Wingless-type MMTV integration site family, member 11	Expressed in metastatic breast cancer with poor survival.	0.0121	[77]
ZEB1	Zinc finger E-box binding Homebox 1	Regulates EMT activation and malignant transformation.	0.0632	[78]

Modulation in expression of genes in MDA231 cells responsible for EMT after treatment with DBMSCs: Differential expression of genes responsible for EMT was observed in MDA231 cells after their treatment with the cellular component of DBMSCs. RT-PCR was performed using the Human Epithelial to Mesenchymal RT^2^ Profiler kit (Qiagen), as explained in the “Material and Methods” section. Assays were run three times, and data were normalized with the untreated control. Data are presented as fold change increase or decrease, calculated from the ΔΔ^−2^ values, and expressed as upregulated or downregulated, in the table.

## Data Availability

The original contributions presented in the study are included in the article/Supplementary Material; further inquiries can be directed to the corresponding author(s).

## Supplementary Materials

The following supporting information can be downloaded at: https://www.mdpi.com/article/10.3390/cells13242131/s1. Figure S1: Standardization of incubation time and dose for treatment of DBMSCs with CM of cancer cells. Figure S2: Migration and invasion of DBMSCs after their treatment with CM of cancer cells. Figure S3: Standardization of dose curve and incubation time for treatment of cancer cells with CM of DBMSCs.
