# Supplementary figures and images for: Partial Inhibition of Epithelial-to-Mesenchymal Transition (EMT) Phenotypes by Placenta-Derived DBMSCs in Human Breast Cancer Cell Lines, In Vitro
Source: Cells. 2024 Dec 23;13(24):2131. doi: 10.3390/cells13242131 (PMC11674051; doi:10.3390/cells13242131)

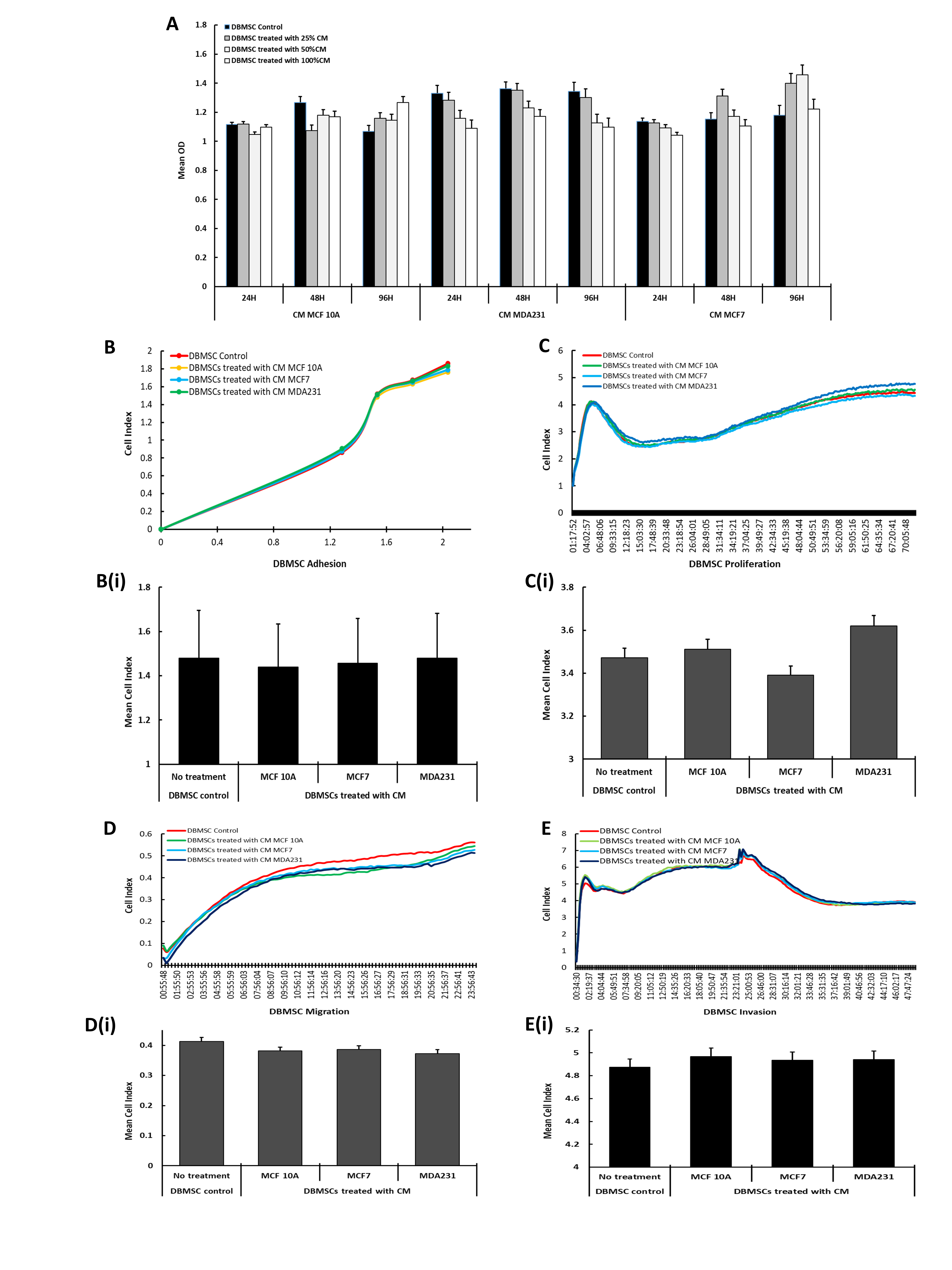

Supplement: Supplementary file 1 [file cells-13-02131-s001.zip › Sup Figure S1.TIF]

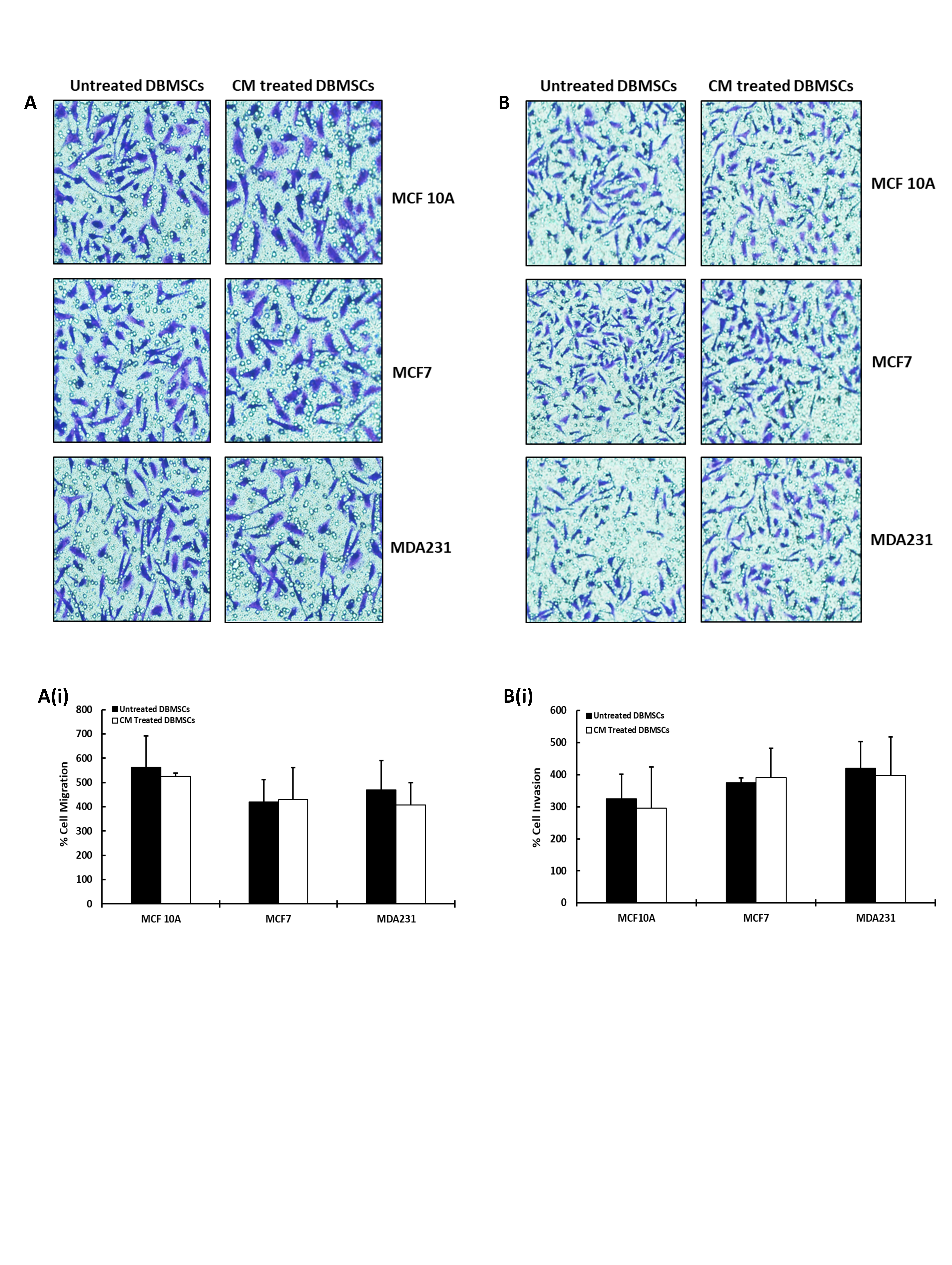

Supplement: Supplementary file 1 [file cells-13-02131-s001.zip › Sup Figure S2.TIF]

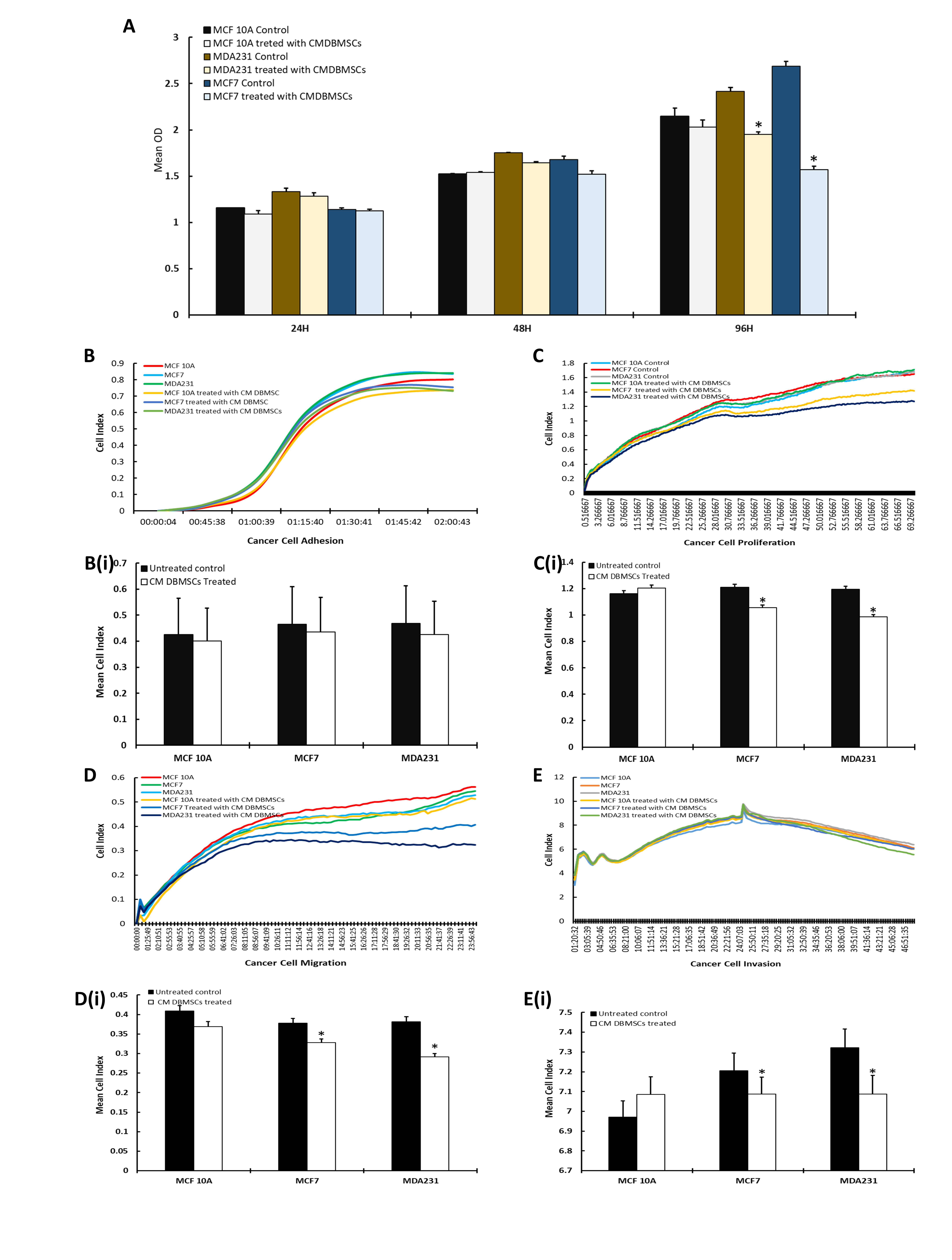

Supplement: Supplementary file 1 [file cells-13-02131-s001.zip › Sup Figure S3.TIF]
